# Practical Dentistry—An Important Movement

**Published:** 1868-02

**Authors:** 


### PRACTICAL DENTISTRY—AN IMPORTANT MOVEMENT.

EDS. BUFFALO COURIER—It may not be generally known by your readers, that there is an effort being made to obtain the enactment of laws for the better regulations of the study and practice of dentistry, with the view of securing a higher standard of qualification, and better dental operations.

This movement originated in Western New York, last October, and has been pushed forward with energy. A delegated Convention made up of representative men in the dental profession, from different parts of the State, was held at Utica, December 17th and 18th. Two days' deliberations resulted in fixing upon the character of a bill, memorial to the legislature, etc., which, together with all other matters to forward the work were entrusted to a committee, consisting of Drs. A. Westcott, of Syracuse; L. W. Rodgers, of Utica, and B. T. Whitney, of Buffalo.

The movement received the hearty endorsement, by special resolution, of the Erie County Medical Society, at its annual meeting, January 14th, and also of the Buffalo Medical Association, at its last meeting.

As this petition explains somewhat the character of the proposed law and its probable good results, it would forward the object by publishing it in the *Courier*.

Yours truly. B. T. WHITNEY.

*To the Legislature of the State of New York:*

The undersigned, your petitioners, would respectfully represent: That while the profession of Dental Surgery is of a great and acknowledged importance to the community at large, not only as a scientific art, but also as an auxiliary branch of general medicine and surgery, requiring for its judicious practice a large amount of artistic and scientific knowledge, the profession is left fully and alike open, and without the least legal mark of distinction to the skillful operator and the unscrupulous charlatan, who has assumed its duties and responsibilities, without the smallest qualification except ignorance and unblushing impudence.

It is left without even recognition by the enactment of a single law pertaining to this profession, which is so eminently calculated to benefit the community and to ameliorate the sufferings of all who require its aid. The natural, and, indeed, necessary consequence is, that while the profession is thus brought into disrepute, the public are made the victims of the grossest impositions by a horde of ignorant pretenders, and too, without the means of discrimination, except by what often proves dearest experience. This state of things prevails to such an extent that thousands at this time are deprived of the blessings which a correct and skillful practice of dentistry is capable of dispensing, simply because they have by such influences been led to doubt the utility of all dental operations by whomsoever performed, all being alike ignored and shunned.

Your petitioners do not ask any prohibitory law, requiring any dentist now in practice to relinquish his already established vocation, but simply the legal organization of State and district societies, similar to those now pertaining to the medical profession, which, while they will be the means of more perfectly disseminating knowledge upon this subject, will also establish some umpire standard of qualification, and which will furnish through their respective diplomas the means by which all meritorious and skillful dentists may be enabled to obtain, and to exhibit to the public, a certificate of the skill which they really possess. Further than this, your petitioners feel that any attempt at interference with the profession as now constituted, would be impolitic, if not unjust.

But as regards those yet to come into the profession, the case stands differently. In respect to these, there can be no good reason why they should not be required by law to pursue a course of study, and for a time commensurate with the importance of this profession, to master which, in all its departments, requires quite as much time and study as to acquire an equally good knowledge of medicine and general surgery.

Your petitioners therefore ask your honorable body to prescribe, by law, a minimum time which shall be devoted to the science and art of Dentistry, before the student shall be admitted to practice. In view of the above considerations, and the fact that an immense amount of comfort or misery hangs upon the decision as to whether the practice of Dentistry shall be in the hands of intelligent and skillful, or left to be dispensed by dishonest quacks, without anything to mark the distinction ; and in view of the fact that over eight millions of dollars are annually expended in this State alone for dental operations, your petitioners deem their prayer as not merely reasonable, but as essential, alike to the welfare of the profession, and the safety of the public at large.

